# A rare case of Endometriosis in vaginal hysterectomy scar

**DOI:** 10.1186/1750-1164-7-6

**Published:** 2013-07-01

**Authors:** Rajiv Mahendru, Sunita Siwach, Deepti Aggarwal, Parveen Rana, Amrita Duhan, Tanya Aggarwal, Tina Anand Mahendru

**Affiliations:** 1 Department of Obs and Gynae, BPS GMC, Khanpur Kalan (Sonepat), Haryana, India; 2 Department of Pathology, BPS GMC, Khanpur Kalan (Sonepat), Haryana, India; 3 Department of Obs and Gynae, MMIMSR, Mullana (Ambala), Haryana, India; 4 Civil Hospital, Ambala, Haryana, India

**Keywords:** Endometriosis, Vaginal Hysterectomy, Vault

## Abstract

Presented hereunder is probably the first reported case of endometriosis at the vaginal apex following vaginal hysterectomy. No other similar case could be traced in the review of the literature.

## Introduction

Scar endometriosis, the presence of ectopic endometrial tissue at scar sites especially following gynecological abdominal surgical procedures like hysterectomy and cesarean section, and in the perineum after vaginal deliveries with episiotomy [1]. Endometriosis, per se, is the presence of such tissue outside of the normal uterine cavity while extrapelvic endometriosis refers to endometriosis found at body sites outside the pelvis [2]. The development of the endometriosis on a surgical scar may have a delayed onset after the surgery and its diagnosis often mistaken for a suture granuloma, incisional hernia, abscess and often predisposes to incorrect diagnosis [3]. Most of the cases reported of scar endometriosis have occurred following obstetrical procedures that exposed the endometrial tissue, especially in cases of cesarean section [4-6]. The term scar endometriosis is used for well-marked fibrous tissue, with thick chocolate-like liquid areas, and is located anywhere in the surgical scar [5]. All scar endometriosis may not be characterized by endometrioma and as such when there are no palpable nodules it is hard to diagnose the disease [6]. The treatment for scar endometriosis is mainly surgical excision of the lesion [7].

## Case report

**Figure 1:**
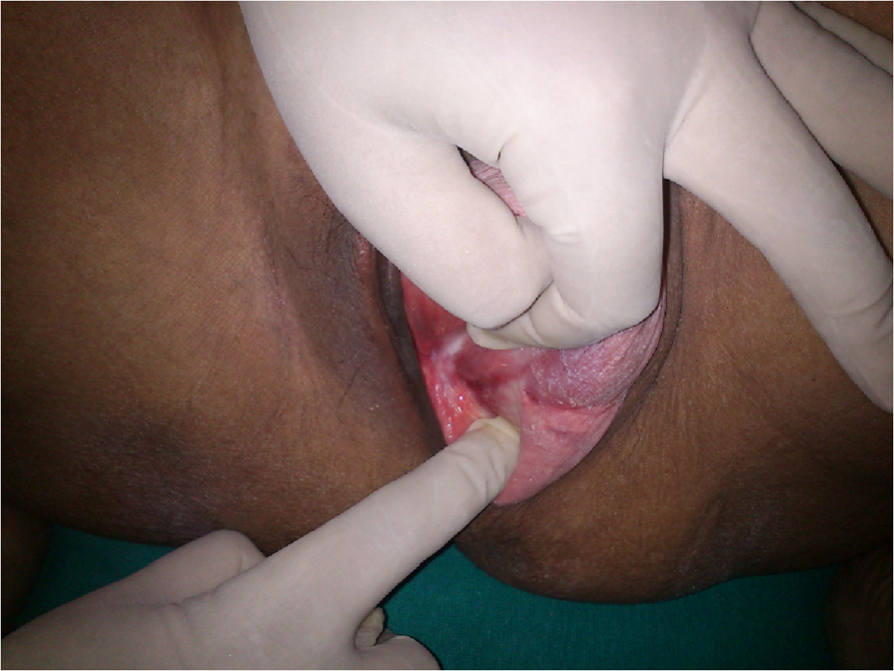
Scar endometriosis as seen at the prolapsed vault.

A 41-year-aged woman presented with a 9 month history of cyclical monthly pain following history of Ward Mayo’s vaginal hysterectomy for genital prolapse approximately two-and –a- half years earlier. Speculum examination revealed a bluish-red discolouration on the right lateral aspect of the prolapsed vault (Figure 1) and on per-vaginal examination a tender swollen area was palpable. On ultrasound examination, an 1.5 × 1.5 cm mixed echogenic nodule with hypoechoic areas enclosed in an area of hyperechogenicity showing diffuse contour was discovered. Routine investigations, including CBC, of the patient were within normal range. She was treated surgically, per abdomen, with excision of the area including 1 cm margin, with the aim of achieving a permanent cure and avoiding locoregional recurrence. Histopathology confirmed the diagnosis with the presence of endometrial glands and stromal cells in the connective tissue.

## Discussion

Scar endometriosis is believed to be the consequence of abdominal fascia or subcutaneous tissue being directly inoculated with endometrial cells during surgical intervention and subsequently stimulated by estrogen during menstrual cycle. In most patients, surgical scar endometriosis involves a painful area that may cyclically swell and become tender before or during the time coinciding with the menses. The real incidence of scar endometriosis is difficult to determine, but is estimated to be as rare as 0.03% to 0.15% with the mean period between the procedure and onset of symptoms being around five years [8]. Failure to close the peritoneum, at the conclusion of gynecological or obstetrical procedures, is postulated to be the reason behind the occurence of scar endometriosis [9]. Medical treatment with the use of progestogens, oral contraceptive pills, and danazol is not effective and gives only partial relief in symptoms and does not ablate the lesion [2]. Excision is the mainstay of treatment of such an entity, and local wide excision to ensure complete removal of the disease is considered to be curative and allays the remote concern for malignant transformation. Local recurrence is likely to be an aftermath of inadequate surgical excision [10]. Moreover, insufficient excision of the lesion leads to the recurrence/renewal of the lesion, making it more extensive and destructive [11].

## Conclusion

Scar endometriosis may be regarded as a differential diagnosis in patients having symptoms of cyclical pain at the incision site not only following gynecological and obsterical abdominal surgeries but even after vaginal hysterectomies.

## Consent

Written informed consent has been obtained from the concerned patient for publication of this Case report and accompanying images. A copy of the written consent is available for review, if required, by the Editor-in-Chief of this journal.

## Competing interest

The authors declare that they have no competing interest.

## Authors’ contributions

RM is the chief surgeon and conceived the manuscript, SS is the co-surgeon and assisted in drafting, DA, PR and AD are the concerned pathologists. TA assisted in data collection, TAM drafted the manuscript. All authors read and approved the final manuscript.
